# Impact of Endometriosis in Women of Arab Ancestry on: Health-Related Quality of Life, Work Productivity, and Diagnostic Delay

**DOI:** 10.3389/fgwh.2021.708410

**Published:** 2021-09-14

**Authors:** Mira Mousa, Moamar Al-Jefout, Habiba Alsafar, Christian M. Becker, Krina T. Zondervan, Nilufer Rahmioglu

**Affiliations:** ^1^Nuffield Department of Women's and Reproductive Health, Endometriosis CaRe Centre, University of Oxford, Oxford, United Kingdom; ^2^Department of Obstetrics and Gynaecology, College of Medical and Health Sciences, United Arab Emirates University, Al Ain, United Arab Emirates; ^3^Department of Obstetrics and Gynaecology, Mutah Medical Faculty, Mutah University, Al-Karak, Jordan; ^4^Center for Biotechnology, Khalifa University of Science and Technology, Abu Dhabi, United Arab Emirates; ^5^Department of Genetics and Molecular Biology, College of Medicine and Health Sciences, Khalifa University of Science and Technology, Abu Dhabi, United Arab Emirates; ^6^Wellcome Centre for Human Genetics, University of Oxford, Oxford, United Kingdom

**Keywords:** quality of life, endometriosis, global health, women's health, Middle East, Arab women, diagnostic delay, work productivity and activity impairment

## Abstract

**Introduction:** Endometriosis has a negative effect on health-related quality of life (HRQoL), wellbeing and daily functioning. Endometriosis is an under-researched condition within non-western populations. Cultural representations are needed to understand the relative roles of societal norms, traditional factors, and religious sensitivities on the impact of endometriosis on HRQoL in various populations. In particular, there is a lack of emphasis placed in understanding the association of HRQoL on endometriosis in Arab women.

**Method:** In this prospective case-control study, 2,610 Arab ancestry women in the United Arab Emirates were recruited to investigate the impact of endometriosis on HRQoL, diagnostic delay, psychological co-morbidities, work productivity, and physical activity. Participants completed the following standardized, validated questionnaires: Short Form-36 version 2 questionnaire, the World Endometriosis Research Foundation EPHect minimum clinical questionnaire version, and Work Productivity and Activity Impairment questionnaire. Translations to the Arabic language, validated using the forward-backward translation method, of the questionnaires were utilized.

**Results:** HRQoL scores were significantly impaired in women with endometriosis, as demonstrated in the Physical Composite Scores and Mental Composite Scores in the symptomatic control group (*p* = 0.001; *p* = 0.003, respectively) and the asymptomatic control group (*p* < 0.001; *p* < 0.001, respectively). Susceptibility and severity of multiple pain syndromes and infertility in women with endometriosis was the main indicator of lower HRQoL. Anxiety (*p* = 0.007) and depression (*p* = 0.005) were significantly associated with endometriosis, in comparison to symptomatic controls. The average diagnostic delay was 11.61 years, however single women experience 15.81 years of diagnosis delay, with approximately 18% (*n* = 15) of the single women experiencing more than a 20-year delay in diagnosis. The intensity of physical activity was not associated with endometriosis, when compared to symptomatic (*p* = 0.405) or asymptomatic controls (*p* = 0.144).

**Conclusion:** For the first time, we provide evidence from a combined hospital, clinic, and population-based study that Arab women with endometriosis experience significant impacts on HRQoL, substantial diagnostic delay after the onset of symptoms, significant association to psychological disorders (anxiety and depression), and a negative impact on work productivity. Future research must focus on understanding the personal and culturally centered beliefs of Arab women to ensure a positive HRQoL trajectory by improving diagnosis and management strategies.

## Introduction

Endometriosis is a chronic gynecological condition that is characterized by endometrium-like tissue located outside the uterine cavity (1). Prevalence rates in the general population are unknown, however, when considering evidence derived from population-based studies of the prevalence of symptoms and findings at laparoscopy, an estimated 10% of premenopausal women have endometriosis, with clinical studies showing that 40–80% of women with endometriosis experience chronic pelvic pain, and 20–50% infertility (1–3). Common symptomatology includes dysmenorrhea, dyspareunia, dyschezia, dysuria, chronic pelvic pain, and infertility (4). Treatment of endometriosis is focused on the amelioration of symptoms, and is limited to repeated surgical removal of lesions, hormonal interventions, such as oral contraceptives, progestin, or GnRH analogs, often with many side effects, and assisted reproduction for infertility (1, 3). The current clinical requirement of surgical visualization to establish a diagnosis means diagnostic delay typically span 7 years (1, 5).

Given the chronic nature of endometriosis, range of pain symptoms, and potential impact on fertility, diagnosis of endometriosis is often delayed, which negatively affects patients' quality of life (5–11). The increased risk of psychological disorders among endometriosis patients interrupts one's regular life, for almost all women in multiple life domains, such as education, work, and intimate relationships (11, 12). The Global Study of Women's Health (GSWH), a cross-sectional study conducted in 10 countries among women undergoing their first laparoscopy for symptoms suggestive of endometriosis, demonstrated a negative impact of endometriosis on HRQoL and work productivity. Absenteeism (*p* = 0.019), presenteeism (*p* = 0.033), and overall work productivity losses (*p* = 0.014) were reported more frequently by women with endometriosis when compared with symptomatic controls (5). Women who experienced longer diagnostic delays reported greater impairment in HRQoL (5). Country-specific differences were observed in the GSWH study, specifically for the duration of diagnostic delay (3–10 years), diagnostic incidence rates (34.8–100%), prevalence of moderate/severe disease stage (American Society of Reproductive Medicine, ASRM, staging; 30–40% in most centers, but nearly 90% in other countries), and monetary loss from endometriosis-associated work absenteeism and presenteeism (varying from US$208 to US$23,712). While the GSWH study included a variety of countries and ethnicities, Middle Eastern countries were not part of the study. Existing research on endometriosis has limited generalizability to the Middle Eastern population where a knowledge gap exists on the differential impact of endometriosis on quality of life. Collecting comprehensive data from all patient populations is critical to understand if there are intrinsic differences in the pathophysiology of endometriosis, in terms of race, genetics, environmental and socioeconomic factors.

In a systematic review with meta-analysis, the pooled prevalence of endometriosis was estimated to be 12.9% (95% CI: 4.2–25.4) in Middle Eastern women undergoing laparoscopy, for any indication (13). While the findings of this review demonstrated that the region lacks reliable, population-based data on the symptomatology and risk factors of endometriosis, there are inherent difficulties in the universal application of symptom and phenotype-based criteria across different countries and cultures, as demonstrated for polycystic ovary syndrome among Arab women (13–17). No studies have assessed the diagnostic delay, work productivity or activity impairment among Middle Eastern women with endometriosis. To the best of our knowledge, only one study utilized a validated quality of life tool (World Health Organization Quality of Life Assessment–BREF) in Middle Eastern women with endometriosis, demonstrating that dysmenorrhea severity is negatively correlated with physical, social and environmental domains (*p* = 0.028; *p* = 0.013; *p* = 0.033, respectively) (18).

Polygamous marriages, strong patriarchal cultures, reticence in disclosing personal matters to the public, and reluctance in seeking care to limit community censorship and victimization is common in the Arab population. These cultural issues limit Arab women's autonomy and may contribute to reproductive and mental health disparities (19–22). In addition, inadequate knowledge on reproductive health literacy and restricted access to mental health services are neglected public health issues in this region, likely to impact women's wellbeing and quality of life (23–25). Given the potential effects of this chronic disease on personal lives, health care systems, and the global community, cross-cultural studies are needed to understand the roles of cultural sensitivities, traditions, and religious norms in the impairment of HRQoL to overall disease burden in Arab women with and without endometriosis pathology.

The Middle Eastern Women Research Association (MAR'A) Project is the first epidemiological study on women's health conditions and related co-morbidities in the Middle East that aims to develop a better understanding of cultural and regional women's health patterns. The findings presented in this study are based on the analyses of data collected within the MAR'A Project, which recruited Arab ancestry women from population and hospital-based settings in the United Arab Emirates (UAE). In this study, we investigated the impact of endometriosis on HRQoL, work productivity, activity impairment and physical activity in Arab ancestry women living in the UAE and evaluated symptoms that may significantly influence HRQoL, adopting the use of standardized, validated questionnaires. Furthermore, we estimated diagnostic delay of endometriosis from onset of symptoms to diagnosis by laparoscopy and prevalence of psychological co-morbidities.

## Methods

### Sample

The Arab region consists of 22 countries located in the Middle East, a transcontinental region that includes the Gulf Cooperative Council region (Bahrain, Kuwait, Oman, Saudi Arabia, Qatar, United Arab Emirates, Yemen), the Levant region (Iraq, Jordan, Lebanon, Palestine, Syria), and the North African region (Algeria, Comoros, Djibouti, Egypt, Libya, Mauritania, Morocco, Somalia, Sudan, and Tunisia) (26). The Arab population are unified by ancestral, ethnic, familial or heritage ties, with common cultural practices, social linguistic influences and traditional norms (27–30).

The UAE is situated in the Middle East, located at the southeastern Arabian Peninsula. Its pivotal geographic position allowed an influx of Arab tribes and foreign expats from the region, providing a representation of the middle eastern population (31). Admixture analysis has shown consistent patterns of the ethnicities and populations of different parts of the Middle East, demonstrating that the contemporary populations of the UAE is suitable to conduct representative epidemiological studies of the Arab population (31, 32).

Ethical approval was obtained from the research ethics committee within the University of Oxford (OXTREC: 25–18) and the Ministry of Health in the UAE (MOHAP/DXB-REC-25/2018). The study followed the General Data Protection Regulation, and adult participants provided written consent to participate in the study after reading the participant information sheet.

### Recruitment and Study Participants

**Figure 1:**
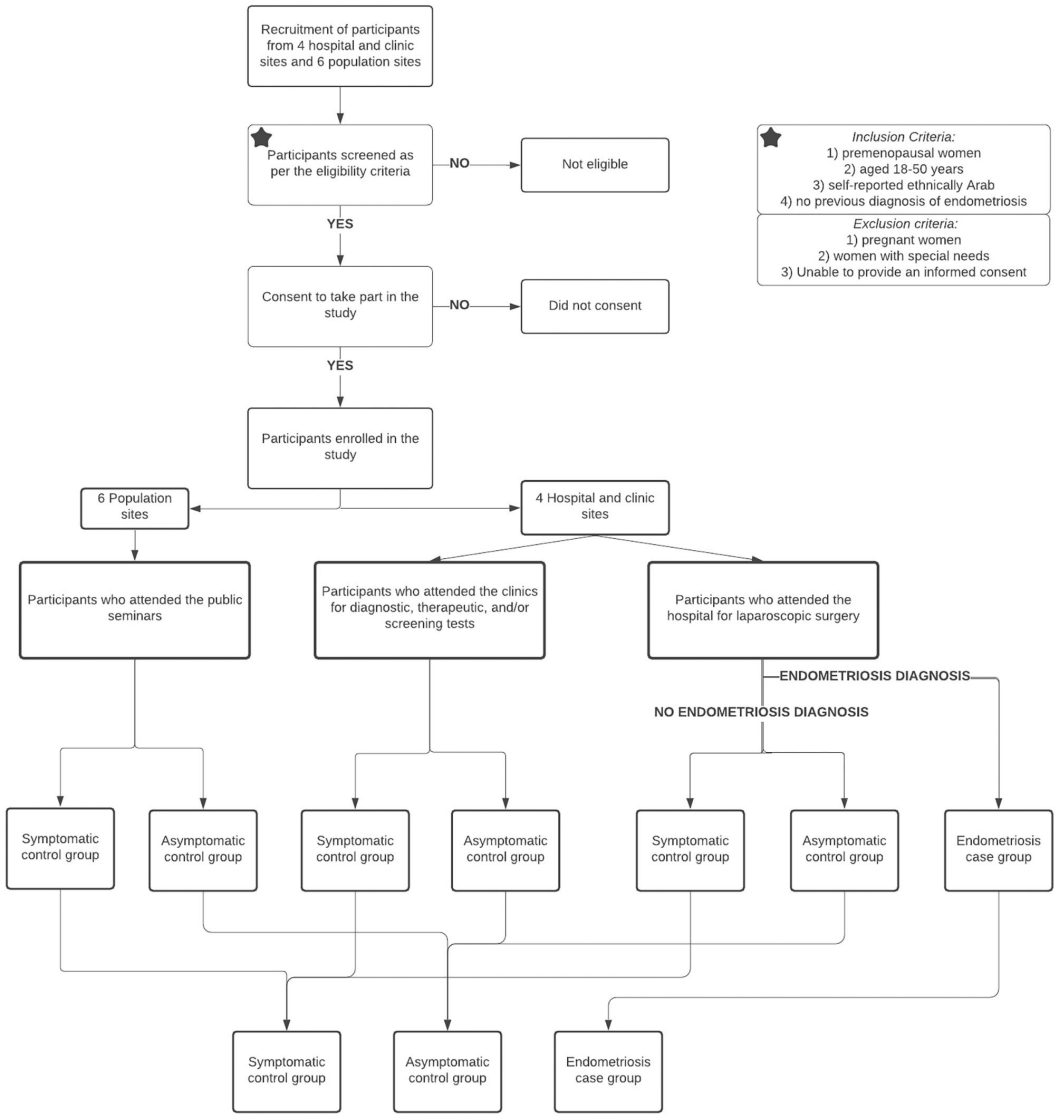
Recruitment flow chart of the study.

The MAR'A Project is a prospective case-control study with multiple recruitment sites across the UAE, including: four hospitals and clinics (women who attended the hospitals for laparoscopic surgery or for diagnostic, therapeutic and/or screening tests), and six population-based sites (women who attended public health seminars organized within the same geographic catchment areas as the participating hospitals and clinics). The participants were approached in the waiting room in the hospitals and clinics, or at the end of the public seminars, and asked if they were interested in learning more about this research study. Between August 2018 and August 2019, we recruited 2,610 participants, using the following criteria: 1) premenopausal women, 2) aged 18–50 years, 3) self-reported ethnically Arab, and 4) no previous diagnosis of endometriosis. Exclusion criteria were: 1) pregnant women, 2) women with special needs, and 3) women who were illiterate, and were unable to comprehend and provide informed consent. Participants who met the inclusion criteria were invited to participate in the study and given the consent form and participant information sheet in their preferred language (English or Arabic), which had been professionally translated using the forward-backward translation methods and approved by the ethics committee. The recruitment flow chart is demonstrated in Figure 1.

The case group were incident endometriosis cases who attended the hospital scheduled to undergo a diagnostic and/or therapeutic laparoscopy or laparotomy for any indication (pelvic pain, subfertility, pelvic mass, adnexal torsion, infection or to be sterilized) by visual confirmation as is the current golden standard (33, 34), and classified into four grades, based on the revised American Society for Reproductive Medicine (rASRM) (35).

The control group comprised three distinct groups: 1) hospital controls who had undergone laparoscopic surgery showing that they were endometriosis-free, 2) clinic controls who were screened using therapeutic/diagnostic tests (such as monitoring for other gynecological conditions or pre-marital screening, ovulation monitoring, cosmetic gynecology, reproductive cancer screening and/or gynecological health check-up), and 3) population controls recruited during organized public seminars. The control groups were sub-divided into symptomatic and asymptomatic controls (Figure 1), to explore sensitivity of results to control definition. Symptomatic controls were defined as: 1) women who experienced chronic pelvic pain, dysmenorrhea, dyspareunia, dyschezia, dysuria for at least 6 months duration with a numerical rating scale (NRS) (36, 37) score greater than four (38), and/or 2) women who were infertile defined as unprotected intercourse in the fertile phase of the menstrual cycles for 12 months (39). Women who did not report the above symptoms were categorized into the asymptomatic control group.

In analysis, comparisons were made between 1) women with endometriosis vs. symptomatic controls, and 2) women with endometriosis vs. asymptomatic controls. The symptomatic control group allowed us to investigate whether observed associations relate to endometriosis *per se*, and not the presence of symptoms. The asymptomatic control group allowed investigation of factors associated with symptoms with or without endometriosis. To quantify hidden bias, such as the possibility of undiagnosed endometriosis cases in the control group, sensitivity analysis was conducted between HRQoL dimensions from the symptomatic controls with surgical confirmation vs. symptomatic controls with no surgical confirmation.

### Questionnaire and Data Collection

Participants completed the following standardized, validated questionnaires and instruments: Short Form-36 version 2 (SF36v2) (40, 41) questionnaire, the World Endometriosis Research Foundation EPHect (WERF-EPHect) minimum clinical questionnaire (EPQ-M) version (42), and Work Productivity and Activity Impairment (WPAI) (43, 44) questionnaire. Translations to the Arabic language, validated using the forward-backward translation method, of the questionnaires were utilized. SF36v2 was used to measure HRQoL and is constructed to measure concepts relating to functional health, well-being, relative burden of the disease, objective and subjective ratings, and self-evaluation of general health status (40, 41). WERF-EPHect questionnaire (EPQ-M) were used to collect demographic information, measure the presence and severity of symptoms, risk factors, phenotypic profile and physical activity assessment (42, 45). WPAI was used to measure absenteeism and presenteeism in employed women, and the impact of symptoms on activities (43, 44).

Data were collected and managed in a web-based research electronic data capture (REDCap) (46) database, with 10% of the questionnaires inputted by two independent data processers to find discrepancies. The following basic anthropometric measurements were collected: (1) weight and height using calibrated digital scaling instruments, and (2) waist and hip circumference using the WHO guidelines (47). After data checking and validation, 2,610 questionnaires were used for data analyses in this study. Pairwise deletion was utilized for variables where data were not missing at random; where reliability of data could not be confirmed by participant, the variable was dropped from the multivariate analysis to minimize the risk of bias.

### Analyses

#### Health Related Quality of Life

The SF-36v2 questionnaire (Cronbach alpha: English language (0.78–0.93) (40, 48–51), Arabic language (0.72–0.94) (41) was analyzed as per the developers' guidelines (40, 41, 48, 50–53). The SF-36 eight dimensions are physical functioning, role limitation-physical, bodily pain, general health, vitality, social functioning, role limitation-emotional, and mental health. Due to the lack of Middle Eastern normative values scores for the general population, the USA population normative values were utilized in this study as a reference population, to allow a meaningful comparison of the data when conducting cross-cultural comparisons (48, 49, 53, 54). The raw score of each of the eight SF-36 dimensions was constructed using the Likert method of summated ratings and each of the eight domains transformed scale was standardized using a z-score transformation. The subscale factor score coefficient for physical component summary (PCS) and mental component summary (MCS) were aggregated using factor analysis. The PCS correlates with physical functioning, role-physical and bodily pain, and the MCS correlates with mental health, role-emotional, and social function. Vitality, general health, and social functioning correlate with both components. T-scores linear transformation is calculated by centering the obtained PCS and MCS sums to get a mean of 50 and a standard deviation (SD) equal to 10 (48, 49). A validated, Arabic translated version was utilized for this study (41).

Means and standard deviations for each of the eight-dimension scales are presented, ranging from 0 to 100, and two composite scores above the normative value (mean of 50 and a SD of 10) demonstrates a healthy QoL. The missing values in the SF-36 dimensions were imputed as recommended by the developer (48, 49): if 50% or more items in one dimension were completed, the mean value of the total completed items were imputed in each missing value; if more than 50% of the items were missing, the dimension score was excluded. In our survey, the item response rate of the 36 items in the SF-36 was 99.77%.

#### Endometriosis Symptomatology, Disease Stage, Comorbidities, and Demographics

The two-composite quality of life scores (PCS and MCS) were compared with pelvic pain severity, presence of endometriosis-associated symptoms, four grade classification based on the revised American Society for Reproductive Medicine (rASRM) (35), and length of time between symptom onset and diagnosis. Pelvic pain severity was reported using the numerical rating scale (NRS) (37), a subjective measure in which individuals rate their pain on an eleven-point numerical scale [score from 0 to 10; Cronbach alpha: English language (0.86–0.95) (37, 55, 56), Arabic language (0.84–0.92) (45)]. Endometriosis-associated symptoms included presence of dysmenorrhea, chronic pelvic pain, dysuria, dyschezia and infertility, and were obtained from the minimal WERF-EPHect questionnaire (EPQ-M). Disease stage was classified according to the rASRM stage 1–4 system using the WERF EPHect minimal surgical form (MSF) and abstracted from the operative notes within the medical records. Participants were asked to report demographic information, past medical history, and lifestyle factors. To assess the impact of endometriosis and associated psychological disorders, participants were asked if they received a clinical diagnosis for anxiety and/or depression.

#### Diagnostic Delay of Endometriosis

Diagnostic delay of endometriosis was quantified by averaging the self-reported earliest age of symptom onset (dysmenorrhea, chronic pelvic pain, dyschezia, dysuria, and infertility) and age of first contact seeking gynecological consultation, and then subtracting from this the age at surgically confirmed diagnosis of endometriosis. Due to societal norms that delay single women from seeking gynecological help, the diagnostic delay of the onset of pelvic pain symptoms was compared between single women and married women. Infertility was not included in the subtype analyses for marital status because single women are prohibited from seeking fertility services.

#### Work Productivity and Activity Impairment

The Work Productivity and Activity Impairment Questionnaire [WPAI; Cronbach alpha: English language (0.77–0.82) (43, 57), Arabic language (0.71–0.76) (44)] is a validated instrument that measures the effect of symptoms and disease on work productivity and daily tasks. The impact of the symptoms is measured by absenteeism (hours of missed work), presenteeism (perceived impairment of work tasks), work productivity loss (perceived loss in productivity levels) and activity impairment (impairment of patient's daily life activities). Higher WPAI percentages indicate a greater impairment and less productivity.

#### Physical Activity Assessment

Physical activity was assessed using a validated measure from the EPQ-M. Participants were asked about their recent physical activity exposure by reporting the average time spent per week during the past year on each of the following activities: walking, jogging, running, bicycling, racquet sports, lap swimming, calisthenics, and other aerobic activity. A metabolic equivalent (MET) score was assigned to each of the eight activities above) to assess the amount of energy the body uses per minute, using the Compendium of Physical Activities (58). The reported minutes per week engaged in each activity was multiplied by the MET score, and the summed value for the individual activities creates the MET/wk. Light-intensity activities are those with <3.0 METs, moderate-intensity activities are those between 3.0 and 6.0 METs, and vigorous-intensity activities score more than 6.0 METs.

### Statistical Methods

The data were analyzed using Statistical Package for Social Science (SPSS) version 20 (SPSS Inc., Chicago, IL) and R (Version 3.4.1). A power calculation determined that a minimum of 1,500 (number of cases = 500; number of controls = 1,000) would need to be recruited to detect an average effect size of 1.15 with a power of 0.8, at a significance level of 0.05. Reporting of the study design was done in adherence to the Strengthening the Reporting of Observational Studies in Epidemiology (STROBE) guidelines (59). Sensitivity analysis was assessed using a non-parametric Wilcoxon Signed Ranks Test to quantify hidden bias between the HRQoL dimensions from the symptomatic controls with surgical confirmation vs. symptomatic controls with no surgical confirmation.

The descriptive variables were verified using frequency analysis. Chi-square and Fisher's exact test were used to study categorical variables. Independent sample *t*-test or nonparametric Mann-Whitney *U*-test were used to study continuous variables. Kruskal-Wallis or ANOVA tests were used to verify the association between the HRQoL domains and the clinical aspects of endometriosis. The chi-squared test was also used to associate the profile of the participants with the staging of endometriosis. Spearman correlation were calculated to establish bivariate relationships between pain severity and HRQoL scores. Multivariate linear regression models tested multivariate relationships between endometriosis-associated symptoms (dysmenorrhea, chronic pelvic pain, dyschezia, dysuria and infertility) and HRQoL scores. Multiple logistic regression analysis was used to study associations between depression and anxiety within the endometriosis and control groups. All regression models accounted for age (continuous), current BMI (underweight, normal weight, overweight, obese), civil status (married, not married), educational status (up to high school level, up to postgraduate level), and work status (working, not working). The significance level adopted for all analyses was *p* ≤ 0.05.

## Results

In total, 3,062 women were approached to participate in this study, however 402 (13.1%) participants declined, leaving 2,660 (86.9%) women in the study. Fifty questionnaires (1.6%) had more than 50% missing data, hence these questionnaires were rejected because they were not completed in line with the study protocol, leaving complete data for 2,610 (85.2%) women. All dyspareunia-related variables were excluded from the analysis, because 96% of the questionnaires had missing data. In view of the conservative social values and cultural beliefs in the region, reluctance among women to report sexual problems was anticipated.

Of the 2,610 women who participated in the study, 518 had surgically-confirmed endometriosis and 2,092 were controls. The recruitment population of the controls were (population-based sample, *n* = 842; hospital-based laparoscopic confirmation, endometriosis-free, *n* = 474; hospital and clinic-based sample, *n* =776), and were grouped according to presence of symptoms [symptomatic control sample (*n* = 1,331); asymptomatic control sample (*n* = 761)]. Most endometriosis cases had rASRM stage I and II endometriosis at the time of their surgical diagnosis [stage I: 129 (24.9%); stage II: 191 (36.8%); stage III: 109 (21.0%); stage IV: 89 (17.2%)].

The mean age of participants was 34.18 (SD ± 7.77) and the mean body mass index (BMI) was 27.00 (SD ± 5.46). Most of the women were married (79.2%, *n* = 2,068), with a higher education (61.3%, *n* = 1,600) and were working (63.6%, *n* = 1,660), which aligns with the annual census data (60). Comparison of the demographic, menstrual history, and prevalence of symptoms in endometriosis cases vs. symptomatic controls and asymptomatic controls is given in Table 1. In comparison to symptomatic and asymptomatic controls, women with endometriosis had a lower BMI (*p* < 0.001; *p* < 0.001), and lower parity (*p* < 0.001; *p* < 0.001). Higher educational achievement and employment rate was not significant between endometriosis cases vs. symptomatic controls (*p* = 0.085; *p* = 0.697). Menstrual irregularity was not associated with endometriosis vs. symptomatic controls (*p* = 0.082); however, a significant difference was present for earlier age at menarche (*p* < 0.001) and longer duration of menstruation (*p* < 0.001). Women with endometriosis had a higher prevalence of being affected by chronic pelvic pain (*p* < 0.001), dysmenorrhea (*p* < 0.001), dysuria (*p* = 0.002), dyschezia (*p* < 0.001), and infertility (*p* = 0.001), in comparison to symptomatic controls. Even with a high prevalence of chronic pain symptoms among the endometriosis cases, the chief clinical complaint and primary reason for surgical investigation was infertility (61.8%, *n* = 320), with only 36.8% (*n* = 191) agreeing to surgical intervention due to their pain symptoms and 1.4% (*n* = 7) proceeding with tubal ligation.

**Table 1:** Demographic details of the participants based on endometriosis cases vs. symptomatic controls and asymptomatic controls.

	**Endometriosis cases (*n* = 518)**	**Symptomatic controls (*n* = 1,331)**	***p*-value**	**Asymptomatic controls (*n* = 761)**	***p*-value**
Age (y) [Mean (SD)]	34.04 (4.81)	35.36 (8.01)	<0.001	32.22 (8.54)	<0.001
Body mass index (kg/m^2^) [mean (SD)]	26.10 (4.55)	27.08 (5.68)	<0.001	27.48 (5.59)	<0.001
Higher education	66.2% (343)	61.9% (824)	0.085	56.9% (433)	0.001
Employment	61.0% (316)	62.0% (825)	0.697	68.2% (519)	0.009
Married	76.3% (395)	79.2% (1,054)	0.169	81.3% (619)	<0.001
Parous	52.1% (270)	62.1% (827)	<0.001	78.6% (598)	<0.001
Parity (Number of children) [mean (SD)]	2.16 (0.94)	2.64 (1.39)	<0.001	3.18 (2.12)	<0.001
Age at Menarche [mean (SD)]	13.12 (1.34)	13.56 (2.57)	<0.001	13.60 (1.19)	<0.001
Menstrual Irregularity	31.3% (162)	27.2% (362)	0.082	20.0% (152)	<0.001
Menstrual Duration [mean (SD)]	5.75 (1.57)	5.35 (1.41)	<0.001	5.51 (1.30)	0.003
Chronic Pelvic Pain	67.0% (347)	57.6% (766)	<0.001	NA	NA
Dysmenorrhea	88.2% (457)	79.0% (1,052)	<0.001	NA	NA
Dysuria	27.0% (140)	16.2% (215)	0.002	NA	NA
Dyschezia	30.1% (156)	18.8% (250)	<0.001	NA	NA
Infertility*	64.9% (281/433)	55.2% (624/1,130)	0.001	NA	NA

NA, Not Applicable, SD: Standard Deviation. *When investigating fertility characteristics, only ever married women (married, widowed, divorced) were included because single women are unable to seek fertility care in the UAE.

### Health-Related Quality of Life

Prior to investigating the impact of HRQoL on endometriosis, a sensitivity analysis was conducted to quantify hidden bias between the HRQoL dimensions of symptomatic controls with laparoscopic confirmation vs. symptomatic controls with no surgical confirmation. The assessment of robustness between the HRQoL dimensions for the two symptomatic controls (surgical investigation vs. no surgical investigation) demonstrated no statistically significant differences in the HRQoL dimensions (physical function, *p* = 0.335; role-physical, *p* = 0.160; body pain, *p* = 0.055; general health, *p* = 0.852; vitality, *p* = 0.615; social functioning, *p* = 0.266; role-emotional, *p* = 0.115; mental health, *p* = 0.862; PCS, *p* = 0.147; MCS, *p* = 0.551), suggesting that results were not dependent on definition of symptomatic control group. Given that we found no change in our effect estimates, this suggests that the potential presence of undiagnosed cases in our symptomatic control group had little impact on the results of the analyses we present.

Compared with symptomatic and asymptomatic women, mean HRQoL scores were significantly lower in the endometriosis case group in all eight dimensions and two composite scores (Table 2). Using the US standardized scores for the calculation of HRQoL, all groups (endometriosis cases, symptomatic controls and asymptomatic controls) had a lower PCS and MCS score than the US normative general population (mean of 50) (49). After adjustment for demographic factors, the HRQoL scores were significantly impaired in women with endometriosis, as demonstrated in the PCS and MCS scores in symptomatic control group (*p* = 0.001; *p* = 0.003, respectively) and asymptomatic control group (*p* < 0.001; *p* < 0.001, respectively).

**Table 2:** Association of endometriosis and HRQoL in comparison to symptomatic controls and asymptomatic controls, measured using the SF36v2 dimensions and composite scores.

	**Endometriosis cases (*n* = 518) Mean (±SD)**	**Symptomatic controls (*n* = 1,331) Mean (±SD)**	**Adjusted *p*-value**	**Asymptomatic controls (*n* = 761) Mean (±SD)**	**Adjusted *p*-value**
Physical function	62.38 (22.59)	66.95 (25.07)	0.001	73.75 (23.40)	<0.001
Role-physical	58.03 (27.06)	64.62 (27.66)	0.001	71.48 (27.65)	<0.001
Body pain	53.77 (29.09)	63.00 (24.35)	<0.001	71.36 (22.15)	<0.001
General health	51.46 (19.41)	55.08 (20.90)	0.003	61.75 (20.63)	<0.001
Vitality	45.15 (17.08)	50.01 (19.07)	0.001	58.88 (20.43)	<0.001
Social functioning	61.12 (23.39)	66.46 (25.11)	0.001	72.66 (24.37)	<0.001
Role emotional	56.39 (22.15)	59.16 (25.49)	0.009	66.55 (25.79)	<0.001
Mental function	52.64 (16.77)	56.58 (17.68)	0.002	64.84 (16.44)	<0.001
PCS	43.60 (8.19)	46.02 (8.43)	0.001	48.28 (7.77)	<0.001
MCS	41.35 (6.83)	42.70 (7.63)	0.003	46.32 (7.43)	<0.001

*MCS, Mental Component Score; PCS, Physical Component Score; SD, Standard Deviation. Adjusted p-values for age (continuous), current BMI (underweight, normal weight, overweight, obese), civil status (married, not married), educational status (up to high school level, up to postgraduate level), and work status (working, not working)*.

There was a significant negative correlation between the subjective NRS rating of pain severity and HRQoL, when conducting a Spearman correlation test. Severity of pelvic pain was inversely correlated with PCS score (correlation coefficient: −0.256, *p* < 0.001) and MCS score (correlation coefficient: −0.116, *p* < 0.001) among endometriosis cases, and among symptomatic controls (correlation coefficient: −0.229, *p* < 0.001, correlation coefficient: −0.136, *p* < 0.001; respectively). However, endometriosis staging was not associated with physical (*p* = 0.815) or mental (*p* = 0.737) component scores, when stratified into the rASRM four-stage system.

After stratifying endometriosis cases and symptomatic controls by pain symptoms (dysmenorrhea, chronic pelvic pain, dyschezia and dysuria) and infertility, and adjusting for demographic factors, all symptomatic women had impaired PCS and MCS, regardless of presence of endometriosis (Table 3). Endometriosis and symptomatic controls could not be distinguished by MCS (Table 3; comparison between B vs. D). This is consistent with other comparative studies that women with and without pathology, matched for presence of pain symptoms, showed no difference on their MCS score (61). However, PCS was significantly lower when comparing the endometriosis cases to symptomatic controls, except for dysuria (*p* = 0.118), which may be because women with endometriosis experience multiple pain symptoms with a greater severity that hinders their physical and daily activities.

**Table 3:** Association between endometriosis symptomatology and SF-36 composite scores for endometriosis cases and symptomatic controls.

	**Endometriosis**		**Symptomatic controls**		**Endometriosis vs. symptomatic controls**	
	PCS (A) Mean* (±SD)	MCS (B) Mean* (±SD)	PCS (C) Mean* (±SD)	MCS (D)Mean* (±SD)	**A vs. C *p*-value**	**B vs. D *p*-value**
Dysmenorrhea	42.93 (8.12)	41.03 (6.83)	44.95 (8.51)	41.74 (7.75)	<0.001	0.090
Chronic Pelvic Pain	42.11 (8.14)	40.71 (6.95)	44.53 (8.51)	41.52 (7.81)	<0.001	0.100
Dyschezia	41.26 (7.32)	40.05 (6.35)	43.11 (7.85)	40.20 (7.28)	0.019	0.862
Dysuria	41.64 (7.08)	40.01 (6.61)	42.89 (7.46)	40.11 (7.01)	0.118	0.428
Infertility	43.38 (8.24)	40.93 (6.75)	45.52 (8.36)	41.63 (7.60)	0.001	0.016

MCS, Mental Component Score; PCS, Physical Component Score; SD, Standard Deviation. *Adjusted mean and p-values for age (continuous), current BMI (underweight, normal weight, overweight, obese), married (married, not married), educational status (up to high school level, up to postgraduate level), and work status (working, not working).

### Diagnostic Delay of Endometriosis

Dysmenorrhea had the earliest symptom onset in the study population, and infertility the latest onset. The delay intervals from the onset of symptoms to first gynecological consultation, and intervals from first gynecological consultation to confirmed diagnosis are depicted in Table 4. Considering the average age at diagnosis was 34.04 (SD: 4.81) years for women with endometriosis, the interval between symptom onset and laparoscopic diagnosis of disease was on average 11.61 (SD: 5.59) years. Within this, the interval from the first onset of symptoms to first gynecological consultation was 6.01 (SD: 4.19) years, and the interval between first gynecological consultation to diagnosis was 6.96 (SD: 4.33) years.

**Table 4:** Symptom onset and diagnostic delay of endometriosis from age at time of diagnosis [years: 34.04 (SD: 4.81)].

			**Delay intervals**		
	**Age at symptom onset (Years) Mean (±SD)**	**Age at first gynecological consultation (Years) Mean (±SD)**	**Onset of symptoms to first gynecological consultation (Years) Mean (±SD)**	**First gynecological consultation to diagnosis (Years) Mean (±SD)**	**Total diagnostic delay (Years) Mean (±SD)**
Dysmenorrhea	17.10 (3.85)	24.02 (5.87)	6.92 (4.86)	9.92 (4.81)	16.83 (5.65)
Chronic Pelvic Pain	20.14 (3.20)	26.57 (5.20)	6.43 (4.20)	7.37 (4.65)	13.80 (5.36)
Dyschezia	23.76 (2.76)	28.92 (4.69)	5.52 (3.98)	5.16 (3.77)	10.33 (5.24)
Dysuria	23.39 (2.80)	28.91 (5.17)	5.16 (3.73)	5.40 (4.10)	10.94 (4.87)
Infertility	NA	26.06 (2.77)	NA	7.32 (4.53)	7.32 (4.53)
Total	21.10 (3.15)	26.90 (5.78)	6.01 (4.19)	6.96 (4.33)	11.61 (5.59)

*NA, Infertility is often diagnosed by a physician, and women are unaware of their age at infertility onset, thus we have included the age at first diagnosis of infertility*.

The delay from symptom onset to diagnosis varied from 7.32 years among women with infertility to 12.44 among those with pelvic pain. Arab women were more likely to seek surgical confirmation of endometriosis when they suffered from infertility, as opposed to pain, hence the shorter diagnostic delay when including infertility confirmation. This is also demonstrated when participants were asked for their chief clinical complaint and primary reason for surgical investigation (infertility, 61.8%; pain, 36.8%).

When investigating the diagnostic delay of pain symptoms, married women experience 10.78 years of delay and single women experience 15.81 years of delay. There was a significant correlation between longer diagnostic delay of endometriosis and reduced HRQoL (PCS: correlation coefficient: −0.233. *p* > 0.001; MCS: correlation coefficient: −0.374, *p* < 0.001).

### Psychological Disorders

Participants were asked to report if they have been clinically diagnosed with anxiety and/or depression disorders. Of endometriosis cases, 28.0% (*n* = 145) reported anxiety, 21.9% (*n* = 292) of symptomatic controls and 6.0% (*n* = 46) of asymptomatic controls. For depression disorder the numbers were 26.3% (*n* = 136), 20.0% (*n* = 266), and 6.6% (*n* = 50), respectively. After adjustment for demographic factors, there was a significant association between endometriosis status both when comparing with symptomatic and asymptomatic controls (Table 5).

**Table 5:** Anxiety and depression in women with endometriosis vs. symptomatic controls and asymptomatic controls.

	**Endometriosis cases vs. Symptomatic controls**				**Endometriosis cases vs. Asymptomatic controls**			
	**Odds ratio (95% CI)**	***p*-value**	**Adjusted odds ratio (95% CI)**	**Adjusted *p*-value**	**Odds ratio (95% CI)**	***p*-value**	**Adjusted odds ratio (95% CI)**	**Adjusted *p*-value**
Anxiety								
No	1.00	0.006	1.00	0.007	1.00	<0.001	1.00	<0.001
Yes	1.40 (1.11, 1.78)		1.38 (1.09, 1.74)		6.04 (4.24, 8.61)		6.46 (4.47, 9.35)	
Depression								
No	1.00	0.003	1.00	0.005	1.00	<0.001	1.00	<0.001
Yes	1.44 (1.15, 1.82)		1.41 (1.11, 1.78)		5.06 (3.57, 7.16)		5.43 (3.77, 7.83)	

*Adjusted odds ratio and p-values for age (continuous), current BMI (underweight, normal weight, overweight, obese), civil status (married, not married), educational status (up to high school level, up to postgraduate level), and work status (working, not working)*.

### Work Productivity and Activity Impairment

Participants were asked to rate the impact of their symptoms on work productivity and daily life using a 10-point scale in the WPAI questionnaire. Most women in the study population were employed (63.6%, *n* = 1,660), and those who did not work were mostly housewives or carers (75.3%; *n* = 716). Of the symptomatic women who were not in employment, approximately one in six (18.0%; *n* = 171) reported that they did not work because of the symptoms for which they underwent surgery. Work productivity was significantly impaired among women in the endometriosis group (Table 6), in comparison to the symptomatic group, for absenteeism (*p* = 0.001), presenteeism (*p* = 0.006), and overall work productivity loss (*p* = 0.002). No significant difference (*p* = 0.059) of impact on non-work-related activities between the endometriosis cases and symptomatic controls was present.

**Table 6:** Work productivity and activity impairment in women with endometriosis and symptomatic controls.

	**Endometriosis cases *n* = 518 %, mean (±SD)**	**Symptomatic controls*n* = 1,331%, mean (±SD)**	**Unadjusted *p*-Value**	**Adjusted *p*-Value**
Absenteeism	16.74 (12.69)	14.76 (12.61)	0.002	0.001
Presenteeism	22.68 (23.06)	18.67 (22.58)	<0.001	0.006
Overall work productivity loss	34.32 (25.11)	29.67 (24.14)	<0.001	0.002
Activity impairment	36.87 (21.56)	33.98 (23.77)	0.008	0.059

*Absenteeism: Time absent from work due to symptoms*.

*Presenteeism: Reduced Effectiveness while on the job due to symptoms*.

*Overall Work Productivity Loss: Combination of Absenteeism and Presenteeism*.

*Activity Impairment: Reduced effectiveness while doing non-work-related activities*.

*Adjusted Odds Ratio and P-Values for age (continuous), current BMI (underweight, normal weight, overweight, obese), civil status (married, not married), educational status (up to high school level, up to postgraduate level), and work status (working, not working)*.

### Physical Activity

The intensity of physical activity was based on a MET score, measured to assess the amount of energy the body uses per minute, using the Compendium of Physical Activities (58). We did not observe any significant association between exercise activity and endometriosis (Table 7). However, all groups (endometriosis cases, symptomatic controls and asymptomatic controls) performed less exercise than the recommended guidelines of at least 500 MET-minutes per week (62). The prevalence of physical inactivity was 67.2% (*n* = 1,754) in the study.

**Table 7:** Physical activity, measured by MET-minutes/week, in women with endometriosis vs. symptomatic controls and asymptomatic controls.

	**Endometriosis cases *n* = 518 Mean (±SD)**	**Symptomatic controls*n* = 1,331Mean (±SD)**	***p*-value**	**Asymptomatic controls*n* = 761Mean (±SD)**	***p*-value**
Light MET	46.68 (44.16)	46.22 (44.16)	0.839	48.49 (45.57)	0.481
Moderate MET	102.85 (183.62)	118.86 (186.07)	0.101	121.46 (201.73)	0.109
Vigorous MET	182.61 (285.08)	186.14 (243.06)	0.790	203.33 (282.97)	0.200
Total MET	332.15 (486.99)	351.22 (423.63)	0.405	373.28 (498.45)	0.144

*MET, Metabolic equivalent*.

*Light-intensity MET: Activities that are those with less than 3.0 METs*.

*Moderate-intensity MET: Activities are those between 3.0 and 6.0 METs*.

*Vigorous-intensity MET: Activities that score more than 6.0 METs*.

## Discussion

For the first time, we provide evidence from a combined hospital, clinic, and population-based study that Arab women with endometriosis experience significant impacts on HRQoL, substantial diagnostic delay after the onset of symptoms, significant association to psychological disorders (anxiety and depression), and a negative impact on work productivity.

All women in the study population (endometriosis, symptomatic controls, and asymptomatic controls) had poorer mental HRQoL than the normative population. To interpret mental health and illness among Arab women, it is important to understand how Arab women perceive their role in their social, cultural, and religious context. The collectivist society emphasizes interdependence, where their role as custodians of community culture is to protect their family from any emotional harm. Emphasis is placed upon family privacy, reputation and solidarity, where women may feel reluctant to seek help due to shame and risk of harming the reputation and honor of the family (22, 63, 64). Therefore, the cultural expectation for Arab women to conceal mental distress may contribute to poor mental HRQoL and increased risk of developing psychological disorders (22, 65).

Physical and mental HRQoL scores were significantly impaired in women with endometriosis, in comparison to symptomatic control group (*p* = 0.001; *p* = 0.003, respectively) and asymptomatic control group (*p* < 0.001; *p* < 0.001, respectively). The effect of endometriosis on physical and mental HRQoL of women was substantial, with SF36v2 scores similar to those reported in Arab women with breast cancer (66). In comparison to the GSWH, the PCS and MCS scores were lower among women of Arab ancestry in endometriosis patients (PCS: 45.1, MCS: 42.2 vs. PCS: 43.6, MCS: 41.3) and symptomatic control group (PCS: 47.6, MCS. 43.5 vs. PCS: 46.0, MCS: 42.7). Susceptibility of multiple pain symptoms and infertility in women with endometriosis was the main indicator of a lower HRQoL. Arab women find it difficult to cope with society, friends, and family's negative reactions to their illness, where they may be blamed for the pain as a sign of punishment or uncontrolled anger, leading to social humiliation and ridicule (20, 64, 67–69).

The impact of pelvic pain severity, with or without surgically confirmed endometriosis pathology, demonstrated a similar direction of effect on PCS score (*p* < 0.001) and MCS score (*p* < 0.0001). The lack of statistical significance in MCS score, when comparing the effect of HRQoL composite scores on endometriosis-associated symptoms, between endometriosis cases and symptomatic controls, demonstrates that the presence, duration and severity of the pain state might be a driving force in the development of sensitization (70, 71). This may suggest that reduced HRQoL is associated with the coexistence of multiple pelvic pain symptoms, as opposed to endometriosis *per se*, although more detailed studies would need to be conducted to relate these causally. Arab women who suffer from chronic pain are subjected to pressure because of the transition from care-provider to care-receiver when suffering from debilitating, symptomatic episodes (72). Due to the burden to their commitments of their roles as mothers and housewives, it may be pain typology and severity that is associated with HRQoL, and not the presence of endometriosis *per se*.

Surgical staging of endometriosis in the present sample was not associated with a difference in the physical (*p* = 0.815) or mental (*p* = 0.737) component scores. This is consistent with previous studies where rASRM surgical staging was not associated with susceptibility or severity of pain or infertility of endometriosis (73). This suggests, perhaps not surprisingly, that the reduction of the HRQoL in women with endometriosis is related to symptomatology rather than rASRM stage.

In this study, only 4% of the women answered questions relating to dyspareunia due to the social taboo of discussing sexual relations and other private affairs. The lack of data in this topic demonstrates that Arab women with sexual pain may suffer in silence, and the impact of psychosocial expectations to prioritize privacy over their quality of life and wellbeing. Further understanding of the type and severity of dyspareunia phenotype may lead to a more personalized treatment approach. To study this sensitive topic among Arab women, sex education and psychosexual therapy must be easily accessible to be able to tackle cultural taboos and social pressures regarding dyspareunia.

Infertility has psychological effects in terms of anxiety, stress, depression and self-esteem, mostly affecting mental health (6). While this association is common across different populations, it may be intensified in this ethnic group because childlessness strongly influences marital life, and unfortunately may lead to divorce, or becoming a second wife in a polygamous marriage (74, 75). Divorced women in Arab societies suffer emotionally and socially, especially when they are infertile. In this study, 88.2% of the divorced women were infertile. Hence, infertility has a significant impact on the mental health components in our study because Arab women are often blamed when conception does not occur, and feel pressure from their husband as if they are not fulfilling their reproductive responsibility (76). This is demonstrated by the particularly low MCS score among infertile women, in both endometriosis and symptomatic control groups.

The diagnostic delay from first onset of symptoms to diagnosis was 11.61 years among Arab women. Within this, the interval from the first onset of symptoms to first gynecological consultation was 6.01 years, and between first gynecological consultation to diagnosis was 6.96 years. The diagnostic delay of 11.61 years among Arab women is approximately 5 years greater than what was reported in the GSWH study (6.7 years), mainly driven by the delay in seeking the first gynecological consultation from symptom onset (6 years among Arab women vs. 1 year in the GSWH study) (5). Due to cultural stigma, it is not common for single women to visit a gynecologist when experiencing chronic pain or menstrual irregularities, hence prolong suffering, as demonstrated in our study where only <20% of the women were single. This is also reflected in the delay of diagnosis by 15.81 years among single women, with approximately 18% (*n* = 15) of the single women experiencing more than a 20-year delay in diagnosis due to chronic pain. Single Arab women were found to have fewer affirming attitudes toward seeking medical care, as they may endanger future marital prospects (77). Additionally, women are often told that their debilitating menstrual pain is normal or unexplained. Hence, the greater delay of diagnosis when suffering from pelvic pain (12.44 years) vs. infertility (7.32 years). Chronic peripheral pain stimulation followed by sensitization of the central nervous system can result in irreversible damage (70, 71). The diagnostic delay that is evident in this study suggests a critical window for attention and early intervention among women with symptoms suggestive of endometriosis. An increased diagnostic delay was associated with poorer HRQoL, due to a longer duration of painful symptoms without an accurate diagnosis and adequate treatment, worsening of the prognosis for fertility and pain, and increasing the risk for repeated operative procedures. The consequence of persistent pain emphasizes that women with endometriosis and symptomatic women with no endometriosis pathology need to consult immediately after symptom onset and be treated actively when they first seek medical care (70, 71, 78–80). This may minimize the development of a state that can cause long-term suffering and reduced HRQoL. Early diagnosis is likely crucial for the effective treatment and prevention of disease progression, as symptoms related to endometriosis can present immediately upon onset of menarche. Arab women may feel reluctant to seek help for their symptoms and sacrifice their own needs and welfare for the sake of the family unit (63, 64). Therefore, in additional to diagnostic delay among the physicians and referral patterns, cultural issues may contribute to the delay in seeking gynecological help.

Anxiety (*p* = 0.007) and depression (*p* = 0.005) were significantly associated with endometriosis, in comparison to symptomatic controls. The overlapping impacts of endometriosis on different aspects of life (physical, psychological, reproductivity, economical, and quality of life), as well as the early onset of symptoms and later diagnosis of the disease, collectively, have a considerable impact on mental health (6, 81, 82). There is a stigma and shame of receiving psychiatric treatment among Arab women to limit community censorship and victimization (77, 83). Rigid cultural norms and expectations oblige Arab women to accept and conceal mental distress and physical pain, which may contribute to an elevated risk of developing psychiatric disorders (77). The prolonged period of the unknown cause of the exacerbating symptoms has a critical impact on a woman's illness trajectory. Endometriosis is a condition that is misdiagnosed, mismanaged, and ignored; in addition to the need for increased health literacy among the population, there is also a lack of proper recognition by health professionals of the symptoms and psychological and interpersonal impact associated with endometriosis. Improving women's health and their HRQoL requires a multi-disciplinary team with a culturally relevant approach to create a suitable environment for providing better psychological care.

Women with endometriosis-related symptoms are negatively affected in a variety of ways relating to work productivity. When comparing the impact of endometriosis-associated symptoms on work productivity and activity impairment, women with endometriosis reported a greater impairment in terms of absenteeism (*p* = 0.001), presenteeism (*p* = 0.006) and overall work productivity loss (*p* = 0.002). However, no significant difference was observed in activity impairment between symptomatic women with and without endometriosis. Greater prevalence of empirical, over-the-counter medication usage by affected women leads to unsuccessful attempts to mitigate this pain, impacting their work productivity (84). Presence and severity of multiple pelvic pain symptoms are the major drivers of work productivity loss in endometriosis (5, 85, 86).

Inconsistent associations were observed between various types of physical activity and endometriosis in published literature (2, 86–89). In this study, the intensity of physical activity was not associated with endometriosis, when compared to symptomatic or asymptomatic controls. The prevalence of physical inactivity in the study was very high, at 67.2%, which aligns with published manuscripts in the region (90–95). There are various personal and environmental factors that act as barriers for Arab women to participate in physical activity: cultural normal and practices of public modesty, lack of accessible environments and fitness facilities for women to feel comfortable to exercise, lack of supportive social milieu for women, conservative dress that is not suitable for physical activity, and lack of information on the importance of physical activity in schools (96, 97). Thus, both general and gender norms are a barrier to physical activity among Arab women, and as such programmes aimed at increasing physical activity are difficult to implement.

Strengths of our study include its large sample size, surgically-confirmed incident endometriosis cases, and the selection of multiple control groups facilitating analyses comparing the contribution of control group selection to the results. Restricting our endometriosis cases to incidental cases diagnosed by laparoscopy decreased misclassification. However, the need for laparoscopy to diagnose endometriosis demonstrates the risk of including women with undiagnosed endometriosis in population-based control groups, resulting in indication biases. Until a non-invasive sample test is available that may be applied at a population level, critical care is needed on the selection of the control group to reduce ascertainment bias. Choosing an appropriate control group for endometriosis cases is complex, and multiple groups sampled from population and hospital based settings, as we used in this study, allow the testing of sensitivity of results to control group choice (98). The control group was recruited from community sites and hospitals/clinics from which the cases were enrolled. Although controls did not report a prior endometriosis diagnosis, some were likely to have undiagnosed endometriosis, meaning our estimates may be affected by misclassification of endometriosis among the controls. This would have driven the associations observed to the null hypothesis of no difference, rather than creating spurious associations. Nevertheless, the prevalence of undiagnosed moderate-severe endometriosis in the general population group is likely to be low (<2%), therefore the control group is unlikely to contain many undiagnosed cases, especially if they were screened for moderate to severe pelvic symptoms (98). In addition, we found no significant magnitude of effect on the distribution of HRQoL dimensions among symptomatic controls (endometriosis-free) with laparoscopic confirmation vs. symptomatic controls with no surgical diagnosis. Women with infertility, or moderate/severe pain symptoms were over-represented in our patient sample because cases were enrolled in hospitals (which are typically more complex and severe cases of endometriosis), when compared to those with asymptomatic or mild symptoms that avoid seeking care from a gynecologist.

In order to compare the quality of life of women with endometriosis with the general population, the official norm-based scores derived from the 1998 USA general population by Quality metric Incorporated were used (49). The reason for choosing this normative population was to allow cross-cultural comparisons, however, the US general population may not be representative of the general Middle Eastern population, for which normative scores have not been published. Higher US scores would lead to a conservative estimation of the decreased quality of life in women with endometriosis. A SF-36 norm-based score must be developed from the Middle Eastern population to utilize for future HRQoL studies. Even though the participants were asked to report clinically diagnoses of anxiety and depression disorders, this information is self-reported and due to the lack of national health registries across the country, we were unable to verify this information. In addition, women are likely to seek treatment from nonpsychiatric specialist, such as general practitioners and gynecologist, for their mental illness due to the associated stigma, therefore this would have driven the associations observed to the null hypothesis (99). HRQoL and symptom severity may be hampered by recall bias because it was collected retrospectively. However, to limit information bias, we restricted our study to women undergoing a first laparoscopy for symptoms suggestive of endometriosis. There are methodological drawbacks with case-control studies, such as uncertain temporal relationships between HRQoL and endometriosis, therefore, a longitudinal cohort study would be more suited to investigate these associations.

It should be noted that the findings may not be generalisable to women who currently reside in countries that are exposed to war (Iraq, Palestine, Syria, Lebanon, Yemen) due to the lack of access to healthcare, low standard of care, low accessibility to adequate medical supplies, lack of qualified medical staff, mental health impact and the lack of attention for women's health due to high mortality and morbidity directly due to war and conflict. In addition, while the study findings may not be generalisable to Arab women residing in countries outside the Middle Eastern region, because they are likely to encounter a different set of social and cultural factors, these findings could be used to make cross-cultural comparisons. For instance, investigating the quality of life of Arab women residing in Western societies can be compared with those in this study, to elucidate similarities and differences among cultures, as well as the genetic and non-genetic underpinnings on HRQoL outcomes. This will increase the likelihood of a positive HRQoL trajectory, improve health and wellness among Arab women, and tackle disparities in the representation of Arabs in endometriosis research, globally. This may also enable an exploration of geographical variations in healthcare provisions for women with endometriosis.

It is important to acknowledge the influence of cultural sensitivities, traditions, and religious heterogeneity in different aspects of reproductive health disparities in healthcare that can act as barriers and directly impact Arab women. HRQoL associated with endometriosis is a growing concern, increasingly voiced by patients and health professionals. Women with endometriosis-associated symptoms should be referred to centers of expertise, led by those with training in complex benign gynecology and referred to a multi-disciplinary team that includes a psychologist, gynecology radiologist, pain specialist and with support from patient organizations. The significantly lower quality of life scores in women with endometriosis and symptomatic control group demonstrate that medical care should also address the emotional and social problems that come with endometriosis. Further research should identify symptom control strategies that target those pathways to improve the outlook for affected women. Our study will enable the establishment of communities for ongoing support and intends to build on the strength of women-centered networks for Arab women.

## Conclusion

The chronicity and severity of pain and infertility associated with endometriosis have a negative impact upon HRQoL, psychological wellbeing and daily functioning. To improve the quality of life in women affected by endometriosis, medical care should address emotional, social and well-being aspects of the disease. The scientific and medical professional need an informed understanding of the heterogenous array of religious beliefs, cultural norms, and traditional expectations in the community, to ensure they are providing culturally sensitive, comprehensive care. There is a need to conduct an in-depth understanding of the personal and culturally centered beliefs of Arab women within the realms of their lives as young adolescents and adults, and how their HRQoL is impacted with endometriosis. Such understanding will allow policymakers, stakeholders, and medical physicians to improve management and develop targeted health education awareness campaigns for Arab women that struggle with endometriosis, which will differ from the current, more western-centric approaches. Additional support is required to target younger Arab women in this region to advance their understanding of reproductive health literacy and increase awareness of the severe impact of this condition and associated symptoms.

## Data Availability Statement

The raw data supporting the conclusions of this article will be made available by the authors, without undue reservation.

## Ethics Statement

The studies involving human participants were reviewed and approved by Research Ethics Committee within the University of Oxford (OXTREC: 25-18) Ministry of Health in the UAE (MOHAP/DXB-REC-25/2018). The patients/participants provided their written informed consent to participate in this study.

## Author Contributions

MM, NR, KZ, and CB were involved in all parts of the study conception, study design, analysis methods, interpretation, drafting of manuscript, and final approval. MA-J and HA were involved in study design, interpretation, reviewing of the manuscript, and final approval. All authors contributed to the article and approved the submitted version.

## Funding

This study was funded by The Sheikh Saud Bin Saqr Al Qasimi Foundation (MR-8180) doctoral grant.

## Conflict of Interest

CB reports grant from Bayer AG, other from AbbVie Inc., grants from Volition Rx, grants from MDNA Life Sciences, grants from Roche Diagnostics Inc., non-financial support from Population Diagnostics Ltd., other from ObsEva, other from Flo Health, outside the submitted work. KZ reports grants from Bayer Healthcare, MDNA Life Sciences, Roche Diagnostics Inc., Volition Rx, and Evotec (Lab282-Partnership programme with Oxford University), all outside the submitted work; and is a Board member (Secretary) of the World Endometriosis Society and World Endometriosis Research Foundation. The remaining authors declare that the research was conducted in the absence of any commercial or financial relationships that could be construed as a potential conflict of interest.

## Publisher's Note

All claims expressed in this article are solely those of the authors and do not necessarily represent those of their affiliated organizations, or those of the publisher, the editors and the reviewers. Any product that may be evaluated in this article, or claim that may be made by its manufacturer, is not guaranteed or endorsed by the publisher.
